# Bridging knowledge translation gap in health in developing countries: visibility, impact and publishing standards in journals from the Eastern Mediterranean

**DOI:** 10.1186/1471-2288-12-66

**Published:** 2012-05-11

**Authors:** Ana Utrobičić, Nauman Chaudhry, Abdul Ghaffar, Ana Marušić

**Affiliations:** 1 Central Medical Library, University of Split School of Medicine, Split, Croatia; 2 Student, University of Michigan, Ann Arbor, USA; 3 Executive Director, Alliance for Health Policy and Systems Research, World Health Organization, Geneva, Switzerland; 4 Department of Research in Biomedicine and Health and Croatian Centre for Global Health, University of Split School of Medicine, Šoltanska 2, 21000, Split, Croatia

## Abstract

**Background:**

Local and regional scientific journals are important factors in bridging gaps in health knowledge translation in low-and middle-income countries. We assessed indexing, citations and publishing standards of journals from the Eastern Mediterranean region.

**Methods:**

For journals from 22 countries in the collection of the Index Medicus for the Eastern Mediterranean Region (IMEMR), we analyzed indexing in bibliographical databases and citations during 2006–2009 to published items in 2006 in Web of Science (WoS) and SCOPUS. Adherence to editorial and publishing standards was assessed using a special checklist.

**Results:**

Out of 419 journals in IMEMR, 19 were indexed in MEDLINE, 23 in WoS and 46 in SCOPUS. Their impact factors ranged from 0.016 to 1.417. For a subset of 175 journals with available tables of contents from 2006, articles published in 2006 from 93 journals received 2068 citations in SCOPUS (23.5% self-citations) and articles in 86 journals received 1579 citations in WoS (24.3% self-citations) during 2006–2009. Citations to articles came mostly from outside of the Eastern Mediterranean region (76.8% in WoS and 75.4% in SCOPUS). Articles receiving highest number of citations presented topics specific for the region. Many journals did not follow editorial and publishing standards, such addressing requirements about the patient’s privacy rights (68.0% out of 244 analyzed), policy on managing conflicts of interest (66.4%), and ethical conduct in clinical and animal research (66.4%).

**Conclusion:**

Journals from the Eastern Mediterranean are visible in and have impact on global scientific community. Coordinated effort of all stakeholders in journal publishing, including researchers, journal editors and owners, policy makers and citation databases, is needed to further promote local journals as windows to the research in the developing world and the doors for valuable regional research to the global scientific community.

## Background

Scientific journals are important tools in knowledge translation from research evidence to practice in health care [1]. However, the majority of the journals publishing health research are concentrated in the developed world, while the gap in knowledge translation in low-and middle-income countries continues to increase [2,3]. A survey of health care providers from 10 low-and middle-income countries about their use of research-based evidence in their practice showed that locally conducted or published research played an important role in changing practice [4]. Furthermore, a survey of researchers from these countries showed that 84% of them had full-text access to at least five scientific journals published locally, nationally or regionally, compared with 59% having access to full-text to at least five scientific journals available through the Health InterNetwork Access to Research Initiative (HINARI) [5]. Another study of funding for the support of knowledge translation in low-and middle-income countries showed that 9 out of 14 national agencies produce or fund local journals [6].

Thus, local scientific journals may have an important role in closing the knowledge translation gap in the developing world, as well as in presenting local research to the mainstream science, i.e. science visible in the major bibliographical and citation indexes [2]. However, while there are a number of reports on research outputs from individual countries or regions [7-12], there is no systematic assessment of the visibility and impact of journals published in low-middle-income countries.

A reliable source of locally produced journals and other health materials in developing countries are the regional indexes kept by the World Health Organization (WHO). One of these indexes covers the Eastern Mediterranean, the region where current social and political changes across the countries have an impact on local health care but are also relevant to global health [13]. The Index Medicus for the Eastern Mediterranean Region (IMEMR) [14] currently includes 521 journals from 22 countries [15]. The aim of our study was to assess the presence of health-related research from the Eastern Mediterranean in the global scientific community. Their visibility was assessed as indexing in major bibliographical databases and their impact as citations to published items in these journals in major global citation databases Web of Science and SCOPUS [16]. We also evaluated the journals’ editorial and publishing standards as an important aspect of quality assurance [17].

## Methods

At the time of the study start in May 2009, the list of journals included in the Index Medicus for the Eastern Mediterranean Region (IMEMR), obtained from the WHO Eastern Mediterranean Regional Office (EMRO) web-site http://www.emro.who.int/library/imjournals/, included 419 journals from the countries in the EMRO region. One journal (*Clinical Diabetes*) contained translations of articles previously published in a US journal with the same title (parent journal) and was not included in the analysis.

Indexing and citation analyses were performed by one of the authors (AU) and independently checked by another (AM). Any disagreements on indexing of or citation to the individual journal or article were discussed between the evaluators and resolved to reach consensus. The assessment of publication standards was performed by one of the authors who had access to the print issues of journals in the WHO EMRO library (NC). The data from the checklists were entered in a database and independently verified by another author (AM) and all inconsistencies were discussed to reach consensus.

### Indexing in bibliographical databases

The indexing of 419 journals listed in IMEMR in May and June 2009 was checked using the following bibliographical/citation databases, available through the subscription by the Croatian Academic and Research Network (CARNet):

1. Academic Search™ Complete – multi-disciplinary full-text bibliographical database from the EBSCO Publishing platform;

2. Agricola, multidisciplinary database from the US National Agricultural Library, available via the OVID platform;

3. CINAHL® – the Cumulative Index to Nursing and Allied Health Literature, from the EBSCO Publishing platform;

4. Current Contents Search® – Full Seven Editions, a current awareness database from the Thomson Reuters, available via the OVID platform;

5. GeoRef – database from the American Geological Institute, available via the EBSCO Publishing platform;

6. PsycINFO®– database from the American Psychological Association, available via the OVID platform;

7. PubMed and MEDLINE from the OVID platform; both databases were analyzed because of slight differences (PubMed but not MEDLINE includes all journals available from PubMedCentral) [18];

8. SCOPUS – citation database from the Elsevier; and

9. Web of Science (WoS) – citation database from the Thomson Reuters.

EMBASE bibliographical database was not separately evaluated because SCOPUS includes EMBASE data and the difference between the two databases is their search engines [19]. The ISSN numbers of journals were verified in the International ISSN Register (http://www.issn.org/).

The indexing was re-checked in June 2010, to ensure the correctness of indexation for 2009.

### Citation analysis

The citation analysis was performed only for journals for which we had available tables of contents from the 2006 volume in print (n = 172 journals). IMEMR could not be used as the source of articles for this analysis as indexing was very often interrupted or incomplete. We analyzed all issues available, including supplements. The name of the first author of each of published articles was the first strategy used to search for citations in the WoS and SCOPUS databases from 2006 to 2009. 2006 was used as a referent year for citation analysis to allow for enough time for the journal items to generate citations. When the name search retrieved more published items, journal name and words from the title were used to narrow the search.

The information on journals’ impact factors and their rankings in subject categories were retrieved from the Journal Citation Reports of the Thomson Reuters [16].

The metrics for journals in the SCOPUS database were retrieved for 2009. Two indicators were retrieved for each journal. SJR (SCImago Journal Rank) is a prestige metrics developed on the basis of SCOPUS database and similar to Google PageRank™ [20]. SJR weighs the citations according to the scientific influence of the journals that generate them. It covers the period of 3 years so that the peak in citations is covered for most journals; it also restricts the self-citations of the journal in inflating the journal’s value. SNIP (Source Normalized Impact per Paper) is the so called ‘contextual impact factor’ because it weighs the citations based on the total number of citations in a subject field, so that the impact of a single citation is given higher value in subject areas where citations are less likely [21].

### Publishing and editorial standards

Publishing and editorial standards were evaluated using the instrument developed by the International Network for the Availability of Scientific Publications (INASP) [17] ( Additional file 1). The instrument is a checklist for assessing editorial and publishing procedures and practices of presenting editorial information and scientific work to ensure that the sources of published data are always identifiable and accessible to readers, and can be properly cited by other authors. We used the first 9 groups of questions from the instrument, assessing the standards in presenting: 1) bibliographical information on the cover page, 2) information about editorial structure, 3) information about the publication inside the journal; 4) information in the “Information for Authors” section, 5) composition of the first page of published articles; 6) structure of published original research articles on medical topics, 7) references and notes; 8) abbreviations, and 9) advertisements. The last two groups of questions (group 10 and 11) of the original checklist were not assessed because they should be answered by someone closely involved with the journal’s editorial and publishing policy. As some items were not applicable to all journals, only the relevant items were evaluated (total 55 items minus the number of items not applicable to a specific journal).

### Statistical analysis

Categorical variables were presented as frequencies, and the data on citations and publication standard items were presented as median and intequartile range (IQR 25% to 75%), or mean and 95% CI, depending on the distribution of data, tested using D’Agostino-Pearson test. Comparison of the percentage of missing items among journals in different databases was performed using a two-tailed Student t-test for 2-group comparisons assuming unequal variances and one-way analysis of variance for multiple group comparisons. All assumptions for the tests were met, and the statistical analysis was performed with help of the MedCalc statistical software (version 10.0.1.0; MedCalc Software, Mariakerke, Belgium).

## Results

### Indexing in bibliographical databases

Indexing of IMEMR journals, as a measure of journal’s visibility, was checked for all 419 journal titles listed in IMEMR at the time of study. The journals had important presence in several analyzed databases (Table 1). The widest journal coverage was in general databases (46 in SCOPUS and 23 in Web of Science). PubMed/MEDLINE, as the specialized health database indexed 19 journals, and other, mostly non-health related databases indexed from 1 to 9 journals (Table 1). The number of indexing years and number of indexed journal items for individual journals are available in Additional file 2: Table S1.

**Table 1:** Indexing of the journals from Index Medicus for the Eastern Mediterranean Region (IMEMR) in major bibliographical databases*

**Database**	**No. of journals indexed**	**No. years of indexation per journal (median, range)**	**Total No. items indexed**	**No. of journal items indexed per journal (median, range)**
SCOPUS	46	8.5 (2–48)	37350	337.5 (52–5247)
Web of Science	23	3 (2–25)	14840	188 (53–6510)
PubMed/MEDLINE	19	11 (3–58)	913	913 (106–5206)
EBSCO-Academic Search	9	3 (2–6)	964	91 (33–230)
CINAHL	4	10 (5–14)	2768	611 (210–1329)
Current Contents	2	17	7794	2372/5422
PsycINFO	1	21	271	271
GeoRef	1	37	183	183

*The analysis was performed in May and June 2009 and verified for 2009 indexation data in June 2010. Journals from WHO EMRO region included in IMEMR: Afghanistan (n = 0), Bahrain (n = 3), Djibouti (n = 0), Egypt (n = 115), Islamic Republic of Iran (n = 104), Iraq (n = 29), Jordan (n = 8), Kuwait (n = 4), Lebanon (n = 8), Libyan Arab Jamahiriya (n = 5), Morocco (n = 6), Oman (n = 2), Pakistan (n63), Palestine (n = 3), Qatar (n = 4), Saudi Arabia (n = 21), Somalia (n = 0), Sudan (n = 9), Syrian Arab Republic (n = 9), Tunisia (n = 8), United Arab Emirates (n = 5), Yemen (n = 7). IMEMR also included journals published in Cyprus (n = 1; *Cyprus Medical Journal*), Germany (n = 3; *Arab Dental*, *Arab Medico*, *Arabmed Journal*), Italy (n = 1; *Clinical Chemistry Newsletter*) and UK (n = 1, *Islamic World Medical Journal*).

#### Language of the journals

Most of the journals were published in English (n = 335, 80.0%), followed by Farsi (n = 49, 11.7%), Arabic (n = 18, 4.3%) and French (n = 14, 3.3%); 2 journals were multilingual (0.5%) and 1 was published in both English and French (0.2%).

#### Length of indexing

In SCOPUS, the journal with the longest uninterrupted indexation was *Saudi Medical Journal*, since 1979. *Lebanese Medical Journal* had the oldest indexation, since 1951, but with interruptions.

The journals with the oldest indexing in WoS were *Saudi Medical Journal* and *Annals of Saudi Medicine* (since 1985) *and Medical Principles and Practice* (since 1993); 20 other journals were recent additions to WoS (2 in 2006, 13 in 2007 and 5 in 2008).

The journal with the longest continuous indexing (since 1981) in PubMed/MEDLINE was the *Journal of the Egyptian Society of Parasitology*, while *Tunisie Medicale* has been indexed since 1949, but with interruptions.

Most of the journals indexed in EBSCO-Academic Search Complete were new additions, with *DARU – Journal of Faculty of Pharmacy Tehran University of Medical Science* as the journal with the longest indexing (since 2004). In EBSCO-CINAHL, *Eastern Mediterranean Health Journal* had the longest uninterrupted indexing (since 1996).

The 2 journals indexed in the Current Contents (*Annals of Saudi Medicine* and *Saudi Medical Journal*) had uninterrupted indexing since 1993 (the first year of database indexing). GeoRef and PsycINFO indexed a single journal each (*Arab Journal of Psychiatry* and *Pakistan Journal of Scientific and Industrial Research*, respectively), since 1966 and 1989, respectively.

Agricola did not have any current indexing of any IMEMR journal, but used to intermittently index 11 journals, the oldest one from 1966 (*Veterinary Medical Journal*). The last year of indexing for this database was 1984 (*Saudi Medical Journal* and *Journal of the Egyptian Society of Parasitology*).

### Citations to journal articles

#### Impact factors and other citation indicators

Out of 23 journals indexed in WoS, 18 were indexed for more than 2 years and had their official impact factor calculated in the Journal Citation Reports database of the Thomson Reuters, while 4 journals had their 5-year impact factors (Table 2). The impact factors ranged from 0.016 for the *Iranian Journal of Veterinary Research* to 1.417 for the *International Journal of Environmental Science and Technology*, also from Iran. Among the journals indexed in SCOPUS (Table 3), the SJR indicator ranged from 0.030 for 2 journals to 0.086 for *Medical Principles and Practice*. SNIP was over 0.300 for 7 journals*: International Journal of Environmental Science and Technology*, *DARU – Journal of Faculty of Pharmacy Tehran University of Medical Sciences*, *Medical Principles and Practice*, *Eastern Mediterranean Health Journal*, *Journal of the College of Physicians and Surgeons Pakistan*, *Iranian Journal of Veterinary Research*, and *Annals of Saudi Medicine* (Table 3).

**Table 2:** Citation metrics for Eastern Mediterranean journals indexed in the Web of Science*

**Journal title**	**Impact factor**	**5-year impact factor**	**JCR category**	**Rank in category/ total journals in category**
*Annals of Saudi Medicine*	0.550	0.505	Medicine, General & Internal	101/133
*Archives of Iranian Medicine*	0.874	–	Medicine, General & Internal	82/133
*Hepatitis Monthly*	0.716	–	Gastroenterology & Hepatology	58/66
*IJPR – Iranian Journal of Pharmaceutical Research*	0.235	–	Pharmacology & Pharmacy	232/237
*International Journal of Environmental Research*	0.781	0.790	Environmental Sciences	150/181
*International Journal of Environmental Science and Technology*	1.417	–	Environmental Sciences	98/184
*Iranian Journal of Allergy, Asthma and Immunology*	0.968	–	Allergy	15/21
			Immunology	116/128
*Iranian Journal of Pediatrics*	0.131	–	Pediatrics	94/94
*Iranian Journal of Public Health*	0.244	–	Public, Environmental & Occupational Health	118/122
*Iranian Journal of Radiation Research*	0.125	–	Radiology, Nuclear Medicine & Medical Imaging	103/104
*Iranian Journal of Reproductive Medicine*	0.183	–	Obstetrics & Gynecology	68/70
*Iranian Journal of Veterinary Research*	0.016	–	Veterinary Sciences	142/142
*IRCMJ – Iranian Red Crescent Medical Journal*	0.071	–	Medicine, General & Internal	131/133
*Medical Principles and Practice*	0.824	0.807	Medicine, General & Internal	85/133
*Neurosciences*	0.112	–	Clinical Neurology	162/167
*Pakistan Journal of Medical Sciences*	0.203	–	Medicine, General & Internal	119/133
*Pakistan Journal of Pharmaceutical Sciences*	0.588	–	Pharmacology & Pharmacy	213/237
*Saudi Medical Journal*	0.510	0.496	Medicine, General & Internal	102/133

*Only journals with 2 or more years of indexation in the Web of Science have official impact factor. 5-year impact factor is available only for journals with more than 5 years of indexing. Source: 2009 Journal Citation Reports, Thomson Reuters.

**Table 3:** Citation metrics for Eastern Mediterranean journals indexed in SCOPUS*

**Journal title**	**SJR**	**SNIP**
*Medical Principles and Practice*	0.086	0.380
*Iranian Journal of Allergy, Asthma and Immunology*	0.084	0.270
*Archives of Iranian Medicine*	0.065	0.280
*IJI – Iranian Journal of Immunology*	0.060	0.160
*Annals of Saudi Medicine*	0.056	0.330
*EMHJ – Eastern Mediterranean Health Journal*	0.055	0.360
*Saudi Medical Journal*	0.054	0.280
*International Journal of Environmental Science and Technology*	0.051	0.460
*IBJ – Iranian Biomedical Journal*	0.051	0.280
*JPMA – Journal of Pakistan Medical Association*	0.051	0.280
*JCPSP – Journal of the College of Physicians and Surgeons Pakistan*	0.050	0.340
*Pakistan Journal of Pharmaceutical Sciences*	0.048	0.150
*DARU – Journal of Faculty of Pharmacy Tehran University of Medical Sciences*	0.043	0.420
*Journal of the Egyptian Society of Parasitology*	0.041	0.150
*Middle East Journal of Anesthesiology*	0.038	0.180
*LMJ – Lebanese Medical Journal*	0.037	0.090
*Iranian Journal of Public Health*	0.036	0.250
*Pakistan Journal of Medical Sciences*	0.036	0.230
*IJMS – Iranian Journal of Medical Sciences*	0.036	0.160
*International Journal of Environmental Research*	0.036	0.070
*Annals of Thoracic Medicine*	0.035	0.170
*Hepatitis Monthly*	0.035	0.090
*International Journal of Diabetes and Metabolism*	0.034	0.190
*Acta Medica Iranica*	0.034	0.090
*JPMI – Journal of Postgraduate Medical Institute*	0.034	0.090
*Neurosciences*	0.034	0.050
*Iranian Journal of Environmental Health Science and Engineering*	0.034	0.040
*JRMS – Journal of Research in Medical Sciences*	0.033	0.100
*Iranian Journal of Pediatrics*	0.033	0.070
*Iranian Journal of Radiation Research*	0.033	0.060
*Saudi Journal of Gastroenterology [The]*	0.033	0.060
*Iranian Journal of Diabetes and Lipid Disorders*	0.033	0.050
*Iranian Journal of Biotechnology*	0.033	0.040
*Iranian Journal of Veterinary Research*	0.031	0.330
*Iranian Journal of Reproductive Medicine*	0.031	0.240
*JLUMHS – Journal of the Liaquat University of Medical Health Sciences*	0.031	0.130
*Pakistan Journal of Scientific and Industrial Research*	0.031	0.100
*Journal of Medicinal Plants*	0.031	0.070
*Iranian Journal of Nuclear Medicine*	0.031	0.040
*JPAD – Journal of Pakistan Association of Dermatologists*	0.031	0.040
*Bahrain Medical Bulletin*	0.030	0.050
*Pan Arab Journal of Neurosurgery*	0.030	0.020
*Emirates Medical Journal*	0.030	0.010
*Medical Forum*	0.030	0.010
*Jordan Medical Journal*	0.029	0.030
*JBMS – Journal of the Bahrain Medical Society*	0.029	0.010

*Abbreviations: SJR – SCImago Journal Rank,^16^ SNIP – Source Normalized Impact per Paper.^17^ For comparison, the 2009 data for *BMC Medical Research Methodology* are SJR = 0.314 and SNIP = 0.93 (2010 impact factor 2.153) and for *Danish Medical Bulletin*, a national journal published in English and in Europe, SJR = 0.305 and SNIP = 0.92 (2010 impact factor 1.600).

In addition to official measures of impact (impact factor, SJR and SNIP), we measured the impact of research published in IMEMR journals by searching for citations to individual published items in citation databases. This analysis was performed for 172 (41.0%) journals for which we had available tables of contents for 2006. For 45 journals (26.2%) we had all available issues, for 123 (71.5%) some issues were missing in the library, and for 4 (2.3%) we could not identify the periodicity of publishing at all. For 6 out of 123 journals with missing issues we could not determine from the information in IMEMR or journals’ websites how many issues had been published in 2006. The journals with clearly identified missing issues lacked 1–11 issues, so that we had available for analysis 10% (n = 1 journal) to 80% (n = 24 journals) of the total publishing output in 2006. For 72 journals we were able to analyze 50% or more of the 2006 publishing output; for 10 journals we also received supplements for 2006. We analyzed all issues available, including supplements, so that the total number of issues was 399 (64.4% out of 620 issues that should have been published in 2006), with 5777 articles.

From 2006 to 2009, 93 journals with articles published in 2006 received citations in SCOPUS, and 85 journals in WoS (journals with more than 10 articles receiving citations in the 2 databases are listed in Additional file 3: Table S2).

#### Citations received by individual articles

For 93 journals that received citations in SCOPUS to their items published in 2006 (n = 4135 items), 979 items (23.7%) were cited in 2006–2009. The total number of citations was 2068; out of those 486 (23.5%) were self-citations. The number of items published in 2006 volumes ranged from 5 to 347 (median 30, IQR 15–54). The number of articles per journal that received citations ranged from 1 to 134 (median 3, IQR 1–7). The median number of total citations per journal was 4 (IQR 1–11.25). The median number of self-citations was 1 (IQR 0–4.25). Most of the journals (52.7%) had 1 to 3 published items that received citations, and most of those (46.2%) received 1 to 3 citations.

For 85 journals that received citations in WoS to their items published in 2006 (n = 4024 items), 817 (20.3%) were cited in 2006–2009. The total number of citations was 1579; out of those 384 (24.3%) were self-citations. The number of items published in 2006 volumes ranged from 5 to 347 (median 31, IQR 16–59). The number of articles per journal that received citations ranged from 1 to 120 (median 2, IQR 1–7). The median number of total citations per journal was 2 (IQR 1–8.25). The median number of self-citations was 1 (IQR 0–4). Most of the journals (61.2%) had 1 to 3 published items that received citations, and most of those (51.8%) received 1 to 3 citations.

Most of the citations to the articles published in the study sample came outside of the Eastern Mediterranean region: 1212 (76.8%) for WoS and 1559 (75.4%) for SCOPUS.

Although the number of journals indexed in the citation databases was small (5.5% of all IMEMR journals for WoS and 11.0% for SCOPUS), the number of journals with citations to their published items was greater, so that 20.3% and 22.2% of all evaluated IMEMR journals received citations in the WoS and SCOPUS, respectively (Table 4). Most of the cited journals were published in Iran (33.6% of all journals from Iran in IMEMR in WoS and 36.5% in SCOPUS) and Pakistan (33.3% of all journals from Pakistan in IMEMR in WoS and 34.9% in SCOPUS) ( Additional file 3: Table S2). The topics of most cited articles were mainly related to local health problems (Table 5). For 3 out of 11 most cited articles in SCOPUS, the citations were almost exclusively self-citations (Table 5). One of them was also almost exclusively self-cited in WoS, and 2 other articles (different from those in SCOPUS) had exclusive self-citations (Table 5).

**Table 4:** Journals from Eastern Mediterranean countries receiving citations to their articles and those being indexed in Web of Science (WoS) and SCOPUS databases*

**Country**	**No. of journals indexed**		**No. journals receiving citation†**		**No. citations (median, IQR)‡**	
	**WoS (n = 23)**	**SCOPUS (n = 46)**	**WoS (n = 85)**	**SCOPUS (n = 93)**	**WoS**	**SCOPUS**
Bahrain	0	2	0	0	0	0
Egypt	0	2	9	9	2.5 (1.0–15.0)	5 (1.7–20.7)
Emirates	0	1	1	1	4	5
Iran	15	22	35	38	4 (1.0–10.0)	3 (1.0–9.0)
Jordan	0	1	1	2	1 / 0	1 / 12
Kuwait	2	1	2	2	3 / 166	7 / 187
Lebanon	0	2	3	3	2 / 3 / 18	1 / 3 / 23
Libya	0	0	2	1	1 / 3	0 / 5
Oman	0	0	1	1	1	5
Pakistan	2	9	21	22	3 (1.0–18.2)	6 (2.0–14.0)
Palestine	0	0	1	1	1	1
Qatar	0	0	2	2	2 / 2	2 / 4
Saudi Arabia	4	6	5	6	15 (8.2–155.2)	15.5 (9.0–131.0)
Sudan	0	0	1	2	0 / 1	1 / 3
Syria	0	0	0	1	0	1
Tunisia	0	0	1	2	0 / 17	1 / 26

*Citations during 2006–2009 to journal articles published in 2006.

†Citations to articles from Eastern Mediterranean country journals regardless of their indexing status in the database.

‡For countries with a small number of journals, citations for the articles are presented for individual journals. IQR – interquartile range (25^th^ – 75^th^ percentile).

**Table 5:** Articles published in Eastern Mediterranean journals in 2006 receiving ≥10 citations in SCOPUS and Web of Science (WoS) during 2006-2009

**SCOPUS**			**WoS**		
**Article**	**Total citations**	**Self citations**	**Article**	**Total citations**	**Self citations**
Al-Maroof, R.A., Al-Sharbatti, S.S. Serum zinc levels in diabetic patients and effect of zinc supplementation on glycemic control of type 2 diabetics (2006) *Saudi Medical Journal*, 27 (3), pp. 344–350.	15	0	Salih, M.A. et al. Study project on stroke in Saudi children. Conclusions, recommendations and acknowledgements. (2006) Saudi Medical Journal, 27 Suppl 1, pp. S108-110.	13	13
Salih, M.A.M. et al. Stroke in Saudi children. Epidemiology, clinical features and risk factors (2006) *Saudi Medical Journal*, 27 (SUPPL.), pp. S12-S20.	14	11	Salih, M.A.M. et al. Stroke in Saudi children. Epidemiology, clinical features and risk factors (2006) Saudi Medical Journal, 27 (SUPPL.), pp. S12-S20.	12	11
Salih, M.A. et al. Study project on stroke in Saudi children. Conclusions, recommendations and acknowledgements. (2006) *Saudi Medical Journal*, 27 Suppl 1, pp. S108-110.	13	13	Al-Maroof, R.A., Al-Sharbatti, S.S. Serum zinc levels in diabetic patients and effect of zinc supplementation on glycemic control of type 2 diabetics (2006) Saudi Medical Journal, 27 (3), pp. 344–350.	11	0
Gaze, D.C. et al. Ischemia-modified albumin concentrations should be interpreted with caution in patients with low serum albumin concentrations (2006) *Medical Principles and Practice*, 15 (4), pp. 322–324.	11	5	Gaze, D.C. et al. Ischemia-modified albumin concentrations should be interpreted with caution in patients with low serum albumin concentrations (2006) *Medical Principles and Practice*, 15 (4), pp. 322–324.	10	5
Weecharangsan, W. et al. Antioxidative and neuroprotective activities of extracts from the fruit hull of mangosteen (Garcinia mangostana Linn.) (2006) *Medical Principles and Practice*, 15 (4), pp. 281–287.	11	1	Amoudy, H.A. et al. Identification of transcriptionally active open reading frames within the RD1 genomic segment of Mycobacterium tuberculosis (2006) *Medical Principles and* *Practice*, 15 (2), pp. 137–144.	10	10
Xiong, G.-S. et al. Role of prophylactic antibiotic administration in severe acute pancreatitis: A meta-analysis (2006) *Medical Principles and Practice*, 15 (2), pp. 106–110.	11	0	Haleem, D.J. Serotonergic modulation of dopamine neurotransmission: A mechanism for enhancing therapeutics in schizophrenia (2006) *Journal of the College of Physicians and Surgeons Pakistan*, 16 (8), pp. 556–562.	10	4
Ayyub, M. et al. Characteristics of dengue fever in a large public hospital, Jeddah, Saudi Arabia. (2006) *Journal of Ayub Medical College*, 18 (2), pp. 9–13.	11	0			
Amoudy, H.A. et al. Identification of transcriptionally active open reading frames within the RD1 genomic segment of Mycobacterium tuberculosis (2006) *Medical Principles and Practice*, 15 (2), pp. 137–144.	10	10			
Irshad, M., Dhar, I. Hepatitis C virus core protein: An update on its molecular biology, cellular functions and clinical implications (2006) *Medical Principles and Practice*, 15 (6), pp. 405–416.	10	0			
Ozgunes, I. et al. The prevalence of extended-spectrum beta lactamase-producing Escherichia coli and Klebsiella pneumoniae in clinical isolates and risk factors (2006) *Saudi Medical Journal*, 27 (5), pp. 608–612.	10	1			
Dashora, K. et al. Effect of processing variables on micro particulate system of aceclofenac. (2006) *Pakistan Journal of Pharmaceutical Science*s, 19 (1), pp. 6–10.	10	5			

During the search for citations, we noticed errors in indexing published items, mostly in pagination, authors’ names and references, and journal or article titles.

### Editorial and publishing standards

Editorial and publishing standards were evaluated in 244 (58.2%) journals available for analysis at the time of the study in 2009 ( Additional file 4: Table S3).

The number of missing items out of 55 evaluation items from 9 questionnaire groups ranged from 2 to 31, with a median of 15 items (IQR 12–18). The percentage of the missing items in relation to total relevant items for a particular journal ranged from 3.8% to 63.8%, with a median of 27.3% (IQR 12.8–35.3%). For 52 journals, some items could not be judged as the relevant information could not be found or was not clear (Table 6). The number of such unclear items for individual journals ranged from 1 to 26 and was mostly due to the language because these journals (40 out of total 46 journals in Farsi and 2 out of 8 journals in Arabic) had unclear or unrecognizable item information. For 7 (2.9%) journals it was difficult to find information on the editorial structure, for 20 (8.2%) the information about the publication, and for 40 (17.5%) the information on the first page of the published article.

**Table 6:** Editorial and publishing standards in Eastern Mediterranean journals (n = 244)*

**Standard (No. evaluation items in category)†**	**No. (%) journals with at least one missing item from the evaluation category**	**No. (%) journals with at least one unclear item from the evaluation category**
Bibliographical information on the cover page (4 items)	66 (27.0)	0
Information about editorial structure (8 items)	238 (97.5)	2 (0.8)
Information about the publication within the journal (9 items)	244 (100.0)	1 (0.4)
Information in the ‘Information for Authors’ section (9 items)	198 (81.1)	48 (19.7)
Composition of the first page of published articles (11 items)	243 (99.6)	7 (2.9)
Structure of published original research (8 items)	4 (1.6)	5 (2.0)
References and notes (4 items)	2 (0.8)	0
Abbreviations (1 items)	0	0
Advertisements (1 items)	0	0

*Country of journal publication: Bahrain (n = 2), Egypt (n = 55), Islamic Republic of Iran (n = 86), Iraq (n = 4), Jordan (n = 5), Kuwait (n = 4), Lebanon (n = 6), Libyan Arab Jamahiriya (n = 3), Morocco (n = 3), Oman (n = 2), Pakistan (n = 41), Palestine (n = 2), Qatar (n = 2), Saudi Arabia (n = 10), Sudan (n = 3), Syrian Arab Republic (n = 3), Tunisia (n = 4), United Arab Emirates (n = 4), Yemen (n = 2), and Germany (n = 1), Italy (n = 1) and UK (n = 1).

†Detailed presentation of data for individual evaluation items is available in ( Additional file 4: Table S3).

#### Missing information in journals

Most of the journals failed to provide important publication information about the journal, including the information about editorial structure, the information about the publication within the journal and the standard information on the first page of published articles (Table 6 and Additional file 4: Table S3). Most often missing item were the statement on the circulation of the journal (96.3% journals), names of the editors responsible for specific sections of the journal (95.1%) and ISSN number (80.7%). ISSN number was missing from the front page of more than a quarter of journals (26.6%) and 50 (20.5%) journals did not display the ISSN anywhere in the journal. In most journals, the first page of the published article did not contain the individual contributions of the authors to the published article (95.1%), sources of funding for the research presented in the manuscript (92.6%), or the dates of manuscript submission and acceptance for publication (61.9%). Guidance for authors of many journals did not address the requirements about the patient’s privacy rights and confidentiality of medical information (68.0%), policy on managing conflicts of interest during publication (66.4%), expectations regarding ethical conduct in clinical and animal research (66.4%), instructions regarding the copyright transfer (45.1%), the process of manuscript evaluation (45.1%) or a clear statement on the scope of the journal (41.0%). The journals also often failed to provide information on the publication itself: the information on subscription (56.6%), list of bibliographical databases where the journal is indexed (55.7%), copyright statement (52.9%), the information on the publisher (48.8%) or the frequency of publication (40.2%).

Journals indexed in bibliographical databases had fewer missing items from the publication standards checklist: journals which were indexed in any of the 3 major databases (MEDLINE, SCOPUS or WoS, n = 52 journals from the analyzed sample) had a mean of 22.9% of missing checklist items (95% CI 21.1 – 24.6%), compared to 30.8% (95% CI 29.4–32.1%) in journals not indexed in these databases (n = 192) (t-test, t = 7.038, *P* < 0.001). As many journals were indexed in all 3 databases ( Additional file 2: Table S1), there were no differences in the mean number of missing items among the journals indexed in the 3 databases: 24.0%, (95% CI 20.0–27.9%) in MEDLINE (n = 19), 23.0% (95% CI 21.4–24.6) in SCOPUS (n = 44), and 20.0% (95% CI 18.1–21.9%) in WoS (n = 20) (ANOVA, F = 2.683, *P* = 0.075).

## Discussion

Our study demonstrated that journals from the countries in the Eastern Mediterranean had an important presence in global science as measured by their visibility – indexing in major bibliographical databases such as MEDLINE, SCOPUS and WoS, and their impact – citations received by articles they published. It is not possible to compare the scientific output of Eastern Mediterranean journals to journals from other world regions, as this is, to the best of our knowledge, the first study assessing regional journals rather than the scientific output of researchers in the region or individual countries.

Citations to articles came predominantly from the journals outside of the Eastern Mediterranean region (76.8% in WoS and 75.4% in SCOPUS), indicating that research published in the Eastern Mediterranean did not generate only local but rather a much wider interest of the scientific community. The number of journals in the Eastern Mediterranean countries (419 in 22 countries) and the volume of articles published by local researchers confirms the finding of previous studies that local journals have important role in bridging the gaps between research and practice in developing countries [4,5]. Our study showed that they also made a contribution to the global research community. Publishing in English (80% of the journals in this study) certainly contributed to more effective communication of research to the global scientific community. Actual circulation of the printed journals could not be assessed because most of the journals (96.3%) did not publish this information. Information about other important editorial and publishing standards, such as requirements about the patient’s privacy rights, policy on managing conflicts of interest, and ethical conduct in clinical and animal research could not be found in more than half of the journals, indicating that IMEMR journals need to work on reaching the excellence in editorial and publishing standards in medical publishing. Indexing in major bibliographical and citation databases was associated with significantly better adherence to publication and editorial standards. This could mean that databases preferentially indexed journals with higher-quality editorial and publishing processes. As we did not follow the editorial and publishing standards in time, it is also possible that indexing in a bibliographical database brought prestige to the journal, which stimulated better editorial and publishing output.

### *Limitations*

The limitations of the study include its cross-sectional design and the availability of the journal for citation and publication standards analysis. While we were able to assess database indexing of all journals in IMEMR, we had access to 41% of the journals for citation analysis and 58% for the assessment of publication standards, reflecting the regularity and completeness of journals’ submission to IMEMR by publishers or editors. As most of the missing data were from small, local journals, the results presented may be an overestimation of the full IMEMR and present only the visibility of the higher-quality journals in IMEMR. Indexing and citation analysis was also limited by the information recorded in the databases and the practices of individual databases. The major problem we encountered was the inconsistency in journal title indexing. Different databases had different variations of the journal titles, mostly because of inconsistent transcription and transliteration, especially in cases when the title included the name of an institution. Another problem for locating individual articles from IMEMR journals in databases was often a significant delay in indexing, which could underestimate the indexing data obtained in this study. Delays may have been the consequence of irregularity in publishing or in submitting published issues to the database, or an omission or delay by a database producer. Another problem specific for IMEMR journals was in locating individual articles using authors’ names because of differences in transcription, transliteration, and indexing of personal names in different databases. The common practice of database producers to index authors by surname and first (and middle) name initial(s) created problems for complex Arabic names, which do not follow the same logic as most western names [22,23]. The final limitation of the study was the fact that the evaluation of publication and editorial standards was inherently subjective in nature and may have influenced the results, especially for journals in Arabic or Farsi languages. Also, the checklist developed by INASP [17] included standards that were not universal for all biomedical journals, such as the requirement to publish authors’ contributions on the first page of a journal article. Many journals, including JAMA, N Eng J Med, Lancet and BMJ [24] publish this information at the end of the article body text, before references. Such variations in publishing styles should be kept in mind in interpreting our results.

### *Problems in reaching global research community*

Although our results cannot be generalized outside of the Eastern Mediterranean, they illustrate common problems of journals from developing areas of the world in reaching the mainstream scientific community. We are not aware of similar comprehensive studies of all journals indexed in large regional index in the developing world, although there are studies of single journals [25] or studies of research outputs from countries [7] in the Eastern Mediterranean; other world regions [9] or individual medical fields [26]; general publication output from countries [27], continents such as Africa [9,10], or developing countries in general [11,12].

### *Implications for stakeholders in scientific publishing*

Despite the limitations of our cross-sectional analysis of IMEMR journals, the results may have important implications for all stakeholders in health research and publishing in the developing world:

1. *Researchers*, both from the Eastern Mediterranean and other parts of the world, should be aware of the importance of the journals in the region and the research they publish. They should not be afraid to publish in good journals from this region or search IMEMR for specific topics unique for Eastern Mediterranean, the region covering 22 countries with different health care systems, unique diseases, environmental issues and sociodemographic specificities [28,29].

2. *Journal editors* could use the metrics from this study to evaluate their own journal in comparison to those that have reached the level of excellence recognized by major international and highly selective databases. They should pay attention to all details of the journal, including the ISSN as perhaps the most important element for the identification of a journal – it should be clearly displayed on the front page and on the masthead page or its equivalent in the electronic edition of the journal. The title should be unique and should be written consistently, as registered with the ISSN registry.Practical advice for journals published in a national language is that they should also clearly register and submit to the indexing databases a parallel official and unique title in English for indexing purposes instead of letting indexing databases resort to free translations. Abbreviations should also be avoided in the titles as they create additional problems because there is no consistency how different databases handle such items.Editors should keep in mind that their work does not end with the indexing into a desired database: they need to periodically make sure that the journal is indexed correctly. Timeliness of indexing, either in IMEMR or international databases, is crucial and is the best way to deal with irregularities and delays in indexing dynamics. The cases we observed where the number of the volume from one year continued to the next year should not happen to journals striving for visibility in and impact on the international scientific community. The quality of the publication and editorial work is also the responsibility of the editor. As our survey showed, publishing excellence can be achieved and maintained, and indexing in international databases is a stimulus for the improvement in adherence to publishing and editorial standards. This requires considerable effort and a high degree of professionalism on the side of journal editors, who are mostly performing their editorial duties on a voluntary basis. However, they can rely on editorial associations and their useful guidelines for good publishing practices [30]. Editors in the Eastern Mediterranean have a particular advantage of very active regional editorial organization, Eastern Mediterranean Association of Medical Editors (EMAME) and should use their collective strength to improve their individual journals [31].

3. *Journal owners*, which are mostly academic institutions or organizations, should recognize their journals as important tools in the knowledge translation for better health care. They should ensure that editors have resources to produce a journal that would be recognized and respected by the local and global scientific community.

4. *National policy makers* could use the methodological approach described in our study to evaluate their national journals and develop their strategies how to best support journals for national and international knowledge translation. National activities could range from establishing national editorial and publishing standards for medical journals and ensuring that journals follow them to the best of their ability, to providing training in different aspects of medical publishing and quality assurance.

5. *International policy makers*, such as WHO, should continue to organize, develop and use locally produced journals and other health materials as a reliable source of information about health in developing countries. For the Eastern Mediterranean Region, good relations with all ministries of health and leading academic and research institutions in the region put the WHO EMRO in a unique position to lead the generation, translation and uptake of research evidence for improved policy and management decisions. Assessment exercises in journal visibility, impact and quality, such as performed in this study, should be repeated every two to five years to follow the development of the journals and develop strategic actions for their improvement.

6. *Producers of indexing databases* should be aware of the specificities of journals from different regions and develop standards for specific issues. They should also collaborate with other stakeholders to ensure that the communication with the scientific journals is not unidirectional, i.e. journals submitting their journal to the database, but to provide the framework for interaction and continual external quality control.

## Conclusions

More collaboration among all stakeholders in research in developing countries is needed to ensure that the traffic on the knowledge translation highway goes both ways, so that the journals from individual countries can continue to be the window to the research in the developing world and the door for valuable regional research to the mainstream scientific community.

## Competing interests

At the time of the study, AG worked in the WHO EMRO in Cairo and NC was a student intern at the same office. The authors have declared that no competing financial interests exist.

## Author contributions

AM and AG conceived the study, AM, AU and NC collected and analyzed the data; all authors contributed to the interpretation of the results; AM wrote the first draft of the paper and AU, NC and AG revised it for intellectual content. All authors agreed on the manuscript submitted to the journal.

## Pre-publication history

The pre-publication history for this paper can be accessed here:

http://www.biomedcentral.com/1471-2288/12/66/prepub

## Supplementary Material

Additional file 1 INASP Evaluation Form. Click here for file

Additional file 2: Table S1 Journals from Index Medicus for the Eastern Mediterranean Region indexed in major bibliographical databases, 2009. Click here for file

Additional file 3: Table S2 Journals with more than 10 articles published in 2006 and cited in Web of Science (WoS) or SCOPUS during 2006-2009. Click here for file

Additional file 4: Table S3 Editorial and publishing standards in Eastern Mediterranean journals (n=244). Click here for file
