# Supplementary material for: Bridging knowledge translation gap in health in developing countries: visibility, impact and publishing standards in journals from the Eastern Mediterranean
Source: BMC Med Res Methodol. 2012 May 11;12:66. doi: 10.1186/1471-2288-12-66 (PMC3430582; doi:10.1186/1471-2288-12-66)
Supplement: Additional file 1 — INASP Evaluation Form. [file 1471-2288-12-66-S1.pdf]

INASP Journal Checklist:

Good practice for editors and publishers

2005

Revised August 2005

This document can be downloaded from the INASP website
http://www.inasp.info/psi/resources.shtml

Developed from the Editorial Good Practice Consensus Document originally prepared by

INTERNATIONAL SCIENTIFIC LITERATURE, INC.
Albertson, NY

MSi
MEDICAL SCIENCE INTERNATIONAL

in collaboration with

International Network INASP
for the Availability of Scientific Publications

Oxford, UK

Index Copernicus

Adopted in Warsaw, 12 July 2004

The Index Copernicus International Scientific Committee
Mark R. Graczynski – Medical Science International, Warsaw, Poland
Marcin Kaminski – Silezian Medical University, Katowice, Poland
Roya Kelishadi - Cardiovascular Research Center, Isfahan, Iran
John Meletis - University of Athens School of Medicine, Greece
Andreas Otte – Germany
Laszlo Rosival - Semmelweis Medical University, Budapest, Hungary
Nahum Sanchez-Mendez
Pippa Smart – International Network for the Availability of Scientific Publications, Oxford, UK
Masayoshi Soma – Japan
Mashalah Torabi – Ministry of Health and Medical Education, Iran
Avi Weinbroum – Israel

The aims of this document

The aim of this Checklist is to provide journal editors and publishers with a simple means of comparing their journal against established norms and so indicate what changes can be made to improve the presentation of their content. Although every journal has its own traditions and personality, standardisation of the way in which editorial information and scholarly work is presented can ensure that the sources of published data are always identifiable and accessible to readers, and can be properly cited by other authors. In addition, high quality editorial and publication practices ensure that articles meet a minimum standard, and are therefore of more value to the development of science.

Workshop exercise

1. Give a copy of your journal, plus this document to another delegate who is unfamiliar with your journal - they will evaluate your journal and return your journal and this document (completed) back to you.
    a. The purpose of having your journal evaluated by somebody else, is that they will not have prior knowledge and will look at the journal without any preconceptions

Self evaluation

Select several issues of your journal, and go through each of the points below, honestly answering the questions, and trying to be as objective as possible.

How to use the document

2. If the journal complies with the criteria, tick the box.
3. If the journal does not comply, put a cross in the box
4. If the criteria is not applicable to the journal - put N/A (Not/Applicable) in the box -or if you do not know, put a ? in the box
5. If you feel a note is required, write it in the margin
6. Some questions are only relevant for biomedical journals – these are indicated by "BM" in the margin

BM	*[b]*	*Guidance on how the journal manages conflicts of interest between referee and author, referee and research sponsor, author and research sponsor, etc.*	

And these items should be disregarded by non-biomedical periodicals.

7. Some questions ask you to judge how easy/difficult it is to locate information - in these cases write in the box "easy" "OK" or "hard" - with any note you feel is helpful to the journal. These questions are indicted with ???? in the margin

????	[m]	How easy is it to locate this information ?	

8. Questions 10 and 11 must be answered by the journal owner/editor/publisher - so for this exercise, the journal and the completed form (up to question 9) should be returned to the delegate who brought this journal to the workshop - so they can answer these questions.

Feedback

The evaluation criteria have been developed through discussion by all the partners and use at INASP workshops, and are subject to infrequent modification. If you have any comments, suggestions or observations, we would be pleased to hear from you: psmart@inasp.info

Journal evaluation

[1]		All the following items should appear clearly on the front cover of each issue of the journal (or on the homepage of the electronic journal):	
	[a]	Journal title	
	[b]	ISSN	
	[c]	Specification of the volume and issue number, and part number if appropriate	
	[d]	Year of publication (plus the month of publication if the journal is a monthly, or the exact date of publication if the journal is a weekly)	
[2]		Within the journal information about editorial structure should be clearly provided, including the following:	
	[a]	The name of the Editor-in-Chief,	
		including affiliation where appropriate, and the town and country where he/she is currently located	
	[b]	The names of the Editorial Board (or panel), etc.	
		with countries where they are located	
	[c]	The names of editors responsible for specific areas – e.g. Book Reviews Editor	
		with the country where located	
	[d]	The name of the editor or administrator responsible for handling receipt of submissions	
	[e]	The correspondence address for the editorial office	
????	[L]	How easy is it to locate this information ?	
[3]		The journal should also clearly provide information about the publication, including the following	
	[d]	The name and address of the publisher	
	[e]	The name of the organisation that sponsors or endorses the publication (if any)	
	[f]	If the journal is available online, this should be stated and the URL of the online version should be given	
	[g]	The journal's print-ISSN (and/or electronic-ISSN if online)	
	[h]	The frequency of publication (monthly, bi-monthly, quarterly, etc.)	
	[I]	The circulation (print/electronic) *this information is rarely given in journals, but it is felt to be useful for authors, sponsors, advertisers, etc., so would prove to be a valuable addition to the journal information*	
	[j]	Information on subscription and single-issue prices, method of payment, etc.	
	[k]	Copyright statement (indicating who owns the overall journal copyright)	
	[L]	All bibliographical indexes and databases where the journal is listed	
????	[m]	How easy is it to locate this information ?	
[4]		A clearly labeled section entitled "Information for Authors" (or the equivalent) should contain the following:<br>*(This need not be included in each issue of the journal if there is pressure for space – but if it does not appear clear instructions on how to find the information should be given.)*	
	[a]	General information on the process of evaluating manuscripts, with a statement of the journal's conformance with international editorial standards and a summary of the peer review process	
BM	*[b]*	*Guidance on how the journal manages conflicts of interest between referee and author, referee and research sponsor, author and research sponsor, etc.*	

BM	*[c]*	*A clear statement of expectations regarding ethical conduct in clinical and animal research*	
BM	*[d]*	*Requirements regarding observance of the patient's privacy rights and confidentiality of medical information*	
	[e]	Instructions regarding assignment of copyright or license-to-publish	
	[f]	The postal address, website and e-mail address for submission or articles (as appropriate), and contact details for any enquiries (telephone, Email, postal)	
	[g]	Clear guidance about the preparation of references (this is one of the most difficult items for authors to prepare correctly, and can undermine the credibility of a good article)	
	[h]	A list of the types of articles the journal seeks to publish	
	[i]	A clear statement of the aims and scope (or remit) of the journal	
????	[L]	How easy is it to locate this information ?	
[5]		One of the most important elements of editorial quality is the uniform composition of the first page of published articles, compatible with the journal's Instructions for Authors. The obligatory elements include:	
	[a]	The full title of the article. This should be no longer than is necessary to convey the gist of the article, while avoiding vagueness or incompleteness, or promising more than the article actually delivers. Journals which publish in local languages should also give the title in English.	
	[b]	The names of all the authors. Whether full first names or simply initials are given depends on the taste and judgment of the editors, but whatever policy is adopted should be applied consistently wherever possible (note: some authors will wish to be listed by their initials only even if full first names are generally given; in the Anglo-Saxon tradition such wishes are always respected).	
BM	*[c]*	*The exact contribution of each co-author, preferably in the following categories: (1) study design: (2) data collection: (3) statistical analysis: (4) literature search: (5) funds collection. No-one should be listed as a co-author who has not made a significant contribution to the work. E.g. the practice of automatically including heads of departments where the research was done (when they were not directly involved in the research), is to be actively discouraged*	
	[d]	The institutional affiliation of each author, if any. Authors without a formal affiliation (e.g. working exclusively in a private practice) should give their city of residence. Academic titles and positions, such as "Prof." or "Department Head," is to be discouraged. At a minimum, the town and country of each author should be given, as this provides valuable information about the source of the article.	
	[e]	Sources of financial support. The name of the supporting institution and grant number should be given. One of the following three headings should be used:<br>[I] "Supported in part by" + name of the supporting institution and grant number<br>[ii] "Departmental sources" – for research supported solely by university/hospital/employment funds<br>[iii] "Self financing" – for research financed privately by authors<br>*(It is not common for this to appear on the opening page, but it is felt to be a useful – and sometimes crucial – piece of information before users read the article.)*	
	[f]	Precise bibliographic reference of the article (the name of the journal, year and volume number, page numbers), so that all copies of the article will contain complete bibliographic information even when detached from the rest of the journal.	
	[g]	The dates when the manuscript was received in the editorial office and when it was accepted for publication. This gives potential authors some idea of the lead time for publication in the journal.	
	[h]	The URL address or DOI (Digital Object Identifier) to the online version [if available].	
	[i]	A correspondence address for one of the authors, preferably with an e-mail address.	
	[j]	A summary of the article (abstract) of 200-250 words. The summary should summarise the	

		main points/findings of the article – it should not be confused with an Introduction. Within medical journals the abstract should be structured, and follow the structure of the article with the exception of the Discussion (Background, Material and methods, Results, Conclusions). An English summary should always be provided for articles published in another language, since frequently only these are indexed by international databases. If an English summary is given, a summary in the local language is optional.	
	[k]	3-6 key words, which should not be words that also occur in the title of the article. (For biomedical articles key words from the MeSH catalogue should be used). Key words should not be adjectives, and should not repeat the title of the article. English key words should always be provided for articles published in another language	
[6] BM		*Original research articles on medical topics should be presented according to the standard format used in medical publishing (with titles and subdivisions within the body of the paper kept to a necessary minimum)*	
BM	*[a]*	*Structured summary (200-250 words, as described in [4.j.] above)*	
BM	*[b]*	*Introduction (or Background). The purpose of the study should be given in the Introduction, not as a separate section.*	
BM	*[c]*	*Materials and methods. The description should be sufficient to allow another researcher to duplicate the experiment.*	
BM	*[d]*	*Results. Sufficient data should be given to allow an independent researcher to verify the results, including statistical analysis. All tables, graphs, photographs and figures should have legends in English (bi-lingual in journals published in other languages).*	
BM	*[e]*	*Discussion. This should also include some remarks on the limitations of the study and suggestions for future research.*	
BM	*[f]*	*Conclusions. Care should be taken not to present as “conclusions” statements that were not proven in the text.*	
BM	*[g]*	*Acknowledgements (if appropriate). Acknowledgement should be regarded as a form of expressing the authors’ gratitude to those institutions or persons who enabled or facilitated the execution of the study, or otherwise made the study feasible, but did not make a personal contribution sufficient to justify co-authorship.*	
BM	*[h]*	*Annex (if appropriate). The Annex may contain detailed descriptions of therapeutic and diagnostic techniques beyond the level of detail needed in the body of the article, samples of test forms and questionnaires used in the study, etc.*	
[7]		References and notes should be treated consistently within each journal	
	[a]	Notes should be kept to a minimum – and the content of the notes should not be extensive. Within a journal, use either footnotes or endnotes and retain a consistency of style	
	[b]	References should be grouped together at the end of the article (it is generally considered old-fashioned to put them in footnotes, and less useful to readers wishing to see the article sources).	
	[c]	References should be presented either in Author/date sequence, or in consecutive order as they are cited in the text (Harvard or Vancouver styles respectively). Citations in the text should be either in the form of author/date or by Arabic numerals respectively. All authors should be listed in the reference, unless there are more than 6 authors, in which case the use of *et al* after the first 3 authors is acceptable.	
	[d]	The references within a journal should all conform to the same style – particularly with regard to the order of the elements (title, volume, date etc.) and the style of the lettering (e.g. italic for journal tiles).	
[8]	[a]	Unless an abbreviation is extremely common, it should be described on the first mention within the text. Where abbreviations are used extensively a list of non-standard abbreviations used in the text should be provided either at the beginning of the article or at the end (before Acknowledgements and References).	
[9]	[a]	Advertisements should be placed on editorial pages (at the beginning and/or at the end of the issue). The placement of advertisements within scientific content (i.e. directly before, inside, or directly after an article) implies a commercial bias in the article, which is to be avoided.	

[10]	[a]	Consistency: it is very important that a journal is consistent in its presentation and editorial style - if it is inconsistent then it looks as if the journal has been assembled carelessly, and the implication is that if the presentation is careless, can the reader believe that the content been prepared carefully?	
	[b]	Style of references:<br>(i) is the reference style consistent within a paper?<br>(ii) Is the reference style consistent throughout a journal issue?	
	[c]	Is the layout of the papers consistent throughout the journal? For example<br>(i) Presentation of lists (numbers, bullets, alignment, etc.)<br>(ii) Use of capitals within text headings and article headings<br>(iii) Alignment, size and typeface of text headings<br>(iv) Typesize and typeface (and single or double column)<br>(v) Presentation of tables: use of lines, alignment of headings (centred, ranged left, etc.)<br>(vi) Author names (full name or initials, order of first and surname)<br>(vii) Presentation of author affiliation/addresses<br>(viii) Running heads within papers<br>(ix) Treatment of quotations (set out, indented, etc.)<br>(x) Figure and table headings (position relative to figure/table, use of bold/italic, alignment, etc.)	
	[d]	Use of symbols and units (for example *P* or p for probability)	
	[e]	Spellings and italicisation	
		**Questions 11 and 12 must be answered by someone who is closely involved with the journal's editorial and publishing policy.**	
[11]	[a]	All articles published within the journal have been reviewed prior to publication, and the review was undertaken by more than one person (i.e. the Editor-in-Chief). Articles are only be published when they fulfill the following criteria:	
	[b]	They are original works and have not been previously published	
	[c]	They contribute to the development of their subject, either by presenting new data, new interpretations or opinions, or new overviews of the topic	
	[d]	The content is not libelous, illegal, or an infringement of anyone’s copyright or other rights	
	[e]	They conform to the requirements of the journal (in particular the moral and ethical requirements) and they comply with the aims and scope of the journal and that their content is appropriate for the readership of the journal	
[12]		In undertaking all activities related to the journal the members of the editorial team undertake to observe professional standards, including	
	[a]	Respecting the confidence of authors (for example regarding patent and legal rights)	
	[b]	Respecting the confidence of reviewers (for example when passing on criticisms of articles to the authors)	
	[c]	Undertaking to process all submitted material in a timely manner, and not to unnecessarily delay any submissions	
	[d]	Undertaking not to abuse the moral rights of the authors who submit to the journal, including misuse of the information they have submitted.	
	[e]	Undertaking not to publish any article until the author(s) have approved the edited version.	
