# Supplementary material for: Bridging knowledge translation gap in health in developing countries: visibility, impact and publishing standards in journals from the Eastern Mediterranean
Source: BMC Med Res Methodol. 2012 May 11;12:66. doi: 10.1186/1471-2288-12-66 (PMC3430582; doi:10.1186/1471-2288-12-66)
Supplement: Additional file 2: Table S1 — Journals from Index Medicus for the Eastern Mediterranean Region indexed in major bibliographical databases, 2009. [file 1471-2288-12-66-S2.doc]

**Table S1.** Journals from Index Medicus of the Eastern Mediterranean Region indexed in major bibliographical databases, 2009

| **Database /Journal title** | **No. years of indexation (median, range)** | **No. of articles indexed (median, range)** |
| --- | --- | --- |
| **SCOPUS:** | **8.5 (2–48)** | **337.5 (52–5247) total=37350** |
| 1. Acta Medica Iranica | 16 | 596 |
| 1. Annals of Saudi Medicine | 22 | 2908 |
| 1. Annals of Thoracic Medicine | 4 | 159 |
| 1. Archives of Iranian Medicine | 8 | 770 |
| 1. Bahrain Medical Bulletin | 14 | 662 |
| 1. DARU – Journal of Faculty of Pharmacy Tehran University of Medical Sciences | 10 | 348 |
| 1. EMHJ – Eastern Mediterranean Health Journal | 14 | 1844 |
| 1. Emirates Medical Journal | 22 | 412 |
| 1. Hepatitis Monthly | 2 | 104 |
| 1. IBJ – Iranian Biomedical Journal | 11 | 291 |
| 1. IJI – Iranian Journal of Immunology | 4 | 124 |
| 1. IJMS – Iranian Journal of Medical Sciences | 24 | 566 |
| 1. International Journal of Diabetes and Metabolism | 9 | 139 |
| 1. International Journal of Environmental Research | 3 | 139 |
| 1. International Journal of Environmental Science and Technology | 5 | 315 |
| 1. Iranian Journal of Allergy, Asthma and Immunology | 5 | 150 |
| 1. Iranian Journal of Biotechnology | 2 | 52 |
| 1. Iranian Journal of Diabetes and Lipid Disorders | 4 | 165 |
| 1. Iranian Journal of Environmental Health Science and Engineering | 3 | 123 |
| 1. Iranian Journal of Nuclear Medicine | 4 | 66 |
| 1. Iranian Journal of Pediatrics | 2 | 145 |
| 1. Iranian Journal of Public Health | 14 | 594 |
| 1. Iranian Journal of Radiation Research | 6 | 164 |
| 1. Iranian Journal of Reproductive Medicine | 2 | 66 |
| 1. Iranian Journal of Veterinary Research | 2 | 126 |
| 1. JBMS – Journal of the Bahrain Medical Society | 21 | 436 |
| 1. JCPSP – Journal of the College of Physicians and Surgeons Pakistan | 14 | 2847 |
| 1. JLUMHS – Journal of the Liaquat University of Medical Health Sciences | 4 | 162 |
| 1. Jordan Medical Journal | 21 | 326 |
| 1. Journal of Medicinal Plants | 6 | 310 |
| 1. Journal of the Egyptian Society of Parasitology | 29 | 2560 |
| 1. JPAD – Journal of Pakistan Association of Dermatologists | 11 | 535 |
| 1. JPMA – Journal of Pakistan Medical Association | 39 | 4760 |
| 1. JPMI – Journal of Postgraduate Medical Institute | 4 | 125 |
| 1. JRMS – Journal of Research in Medical Sciences | 5 | 353 |
| 1. LMJ – Lebanese Medical Journal | 48 | 1955 |
| 1. Medical Forum | 10 | 760 |
| 1. Medical Principles and Practice | 16 | 1036 |
| 1. Middle East Journal of Anesthesiology | 34 | 1090 |
| 1. Neurosciences | 8 | 657 |
| 1. Pakistan Journal of Medical Sciences | 10 | 1156 |
| 1. Pakistan Journal of Pharmaceutical Sciences | 5 | 327 |
| 1. Pakistan Journal of Scientific and Industrial Research | 31 | 1232 |
| 1. Pan Arab Journal of Neurosurgery | 4 | 232 |
| 1. Saudi Journal of Gastroenterology [The] | 4 | 216 |
| 1. Saudi Medical Journal | 31 | 5247 |
| **Web of Science:** | **3 (2–25)** | **188 (53–6510) total=14840** |
| 1. Annals of Saudi Medicine | 25 | 3266 |
| 1. Annals of Thoracic Medicine | 2 | 85 |
| 1. Archives of Iranian Medicine | 3 | 317 |
| 1. Hepatitis Monthly | 3 | 144 |
| 1. IJPR – Iranian Journal of Pharmaceutical Research | 3 | 124 |
| 1. International Journal of Environmental Research | 3 | 179 |
| 1. International Journal of Environmental Science and Technology | 3 | 208 |
| 1. Iranian Journal of Allergy, Asthma and Immunology | 3 | 100 |
| 1. Iranian Journal of Parasitology | 2 | 76 |
| 1. Iranian Journal of Pediatrics | 3 | 234 |
| 1. Iranian Journal of Public Health | 4 | 341 |
| 1. Iranian Journal of Radiation Research | 3 | 351 |
| 1. Iranian Journal of Reproductive Medicine | 3 | 107 |
| 1. Iranian Journal of Veterinary Research | 3 | 188 |
| 1. IRCMJ – Iranian Red Crescent Medical Journal | 3 | 214 |
| 1. JRMS – Journal of Research in Medical Sciences | 2 | 121 |
| 1. KMJ – Kuwait Medical Journal | 2 | 130 |
| 1. Medical Principles and Practice | 16 | 900 |
| 1. Neurosciences | 4 | 363 |
| 1. Pakistan Journal of Medical Sciences | 3 | 656 |
| 1. Pakistan Journal of Pharmaceutical Sciences | 3 | 173 |
| 1. Saudi Medical Journal | 25 | 6510 |
| 1. Yakhteh | 3 | 53 |
| **PubMed/MEDLINE:** | **11 (3–58)** | **913 (106–5206) total=30003** |
| 1. Annals of Saudi Medicine | 19 | 2791 |
| 1. Archives of Iranian Medicine | 4 | 462 |
| 1. EMHJ – Eastern Mediterranean Health Journal | 11 | 1595 |
| 1. IBJ – Iranian Biomedical Journal | 3 | 106 |
| 1. IJI – Iranian Journal of Immunology | 4 | 107 |
| 1. IJKD – Iranian Journal of Kidney Diseases | 3 | 122 |
| 1. Iranian Journal of Allergy, Asthma and Immunology | 7 | 237 |
| 1. JAMC – Journal of Ayub Medical College – Abbotabad – Pakistan | 9 | 767 |
| 1. JCPSP – Journal of the College of Physicians and Surgeons Pakistan | 8 | 1792 |
| 1. Journal of the Egyptian Public Health Association [The] | 38 | 1195 |
| 1. Journal of the Egyptian Society of Parasitology | 29 | 2591 |
| 1. JPMA – Journal of Pakistan Medical Association | 38 | 4579 |
| 1. LMJ – Lebanese Medical Journal | 48 | 1999 |
| 1. Medical Principles and Practice | 8 | 663 |
| 1. Middle East Journal of Anesthesiology | 27 | 913 |
| 1. Pakistan Journal of Pharmaceutical Sciences | 22 | 646 |
| 1. Saudi Medical Journal | 11 | 3889 |
| 1. Tunisie Medicale [La] | 58 | 5206 |
| 1. Urology Journal | 6 | 343 |
| **EBSCO – Academic Search Complete:** | **3 (2–6)** | **91 (33–230) total=964** |
| 1. Annals of Thoracic Medicine | 4 | 166 |
| 1. DARU – Journal of Faculty of Pharmacy Tehran University of Medical Sciences | 6 | 230 |
| 1. Egyptian Journal of Hospital Medicine [The] | 3 | 108 |
| 1. Egyptian Journal of Medical Human Genetics [The] | 2 | 35 |
| 1. Hepatitis Monthly | 2 | 58 |
| 1. Iranian Journal of Nuclear Medicine | 3 | 33 |
| 1. Iranian Journal of Pediatrics | 3 | 74 |
| 1. Journal of Mazandaran University of Medical Sciences | 2 | 91 |
| 1. Saudi Journal of Gastroenterology [The] | 3 | 169 |
| **EBSCO– CINAHL:** | **20 (5–14)** | **611 (210–1329)**  **total=2768** |
| Annals of Saudi Medicine | 12 | 949 |
| EMHJ – Eastern Mediterranean Health Journal | 14 | 1328 |
| HAYAT – Journal of Faculty of Nursing and Midwifery [The] | 8 | 210 |
| Journal of Islamic Dental Association of Iran [The] | 5 | 273 |
| **Current Contents (all editions):** |  | **total=7794** |
| 1. Annals of Saudi Medicine | 17 | 2372 |
| 1. Saudi Medical Journal | 17 | 5422 |
| **PsychINFO:** | | |
| 1. Arab Journal of Psychiatry [The] | 21 | 271 |
| **GeoRef:** | | |
| 1. Pakistan Journal of Scientific and Industrial Research | 37 | 183 |
| **Index Copernicus:** | | |
| 1. Armaghane-danesh | | |
| 1. ARYA Atherosclerosis | | |
| 1. Audiology | | |
| 1. Bina Journal of Ophthalmology | | |
| 1. Blood | | |
| 1. Bulletin of Alexandria Faculty of Medicine | | |
| 1. Bulletin of Faculty of Pharmacy, Cairo University | | |
| 1. Bulletin of High Institute of Public Health [The] | | |
| 1. Bulletin of Pharmaceutical Sciences – Assiut University | | |
| 1. Bulletin of the National Research Centre | | |
| 1. Bulletin of the Ophthalmological Society of Egypt | | |
| 1. DARU – Journal of Faculty of Pharmacy Tehran University of Medical Sciences | | |
| 1. DRJ – Dental Research Journal | | |
| 1. EDJ – Egyptian Dental Journal | | |
| 1. Egyptian Journal of Biophysics and Biomedical Engineering | | |
| 1. Egyptian Journal of Community Medicine [The] | | |
| 1. Egyptian Journal of Dermatology and Andrology | | |
| 1. Egyptian Journal of Histology [The] | | |
| 1. Egyptian Journal of Hospital Medicine [The] | | |
| 1. Egyptian Journal of Medical Human Genetics [The] | | |
| 1. Egyptian Journal of Medical Laboratory Sciences | | |
| 1. Egyptian Journal of Microbiology | | |
| 1. Egyptian Journal of Nutrition | | |
| 1. Egyptian Journal of Occupational Medicine | | |
| 1. Egyptian Journal of Pharmaceutical Sciences | | |
| 1. Egyptian Journal of Psychiatry [The] | | |
| 1. Egyptian Journal of Surgery [The] | | |
| 1. Egyptian Journal of Urology | | |
| 1. Egyptian Medical Journal of the National Research Center | | |
| 1. Egyptian Orthopaedic Journal [The] | | |
| 1. Egyptian Pharmaceutical Journal [ | | |
| 1. Egyptian Rheumatology and Rehabilitation | | |
| 1. Egyptian Science Magazine [The] | | |
| 1. EJB – Egyptian Journal of Biochemistry and Molecular Biology [The] | | |
| 1. EMHJ – Eastern Mediterranean Health Journal | | |
| 1. Gazette of the Egyptian Paediatric Association [The] | | |
| 1. Govaresh | | |
| 1. HAKIM Research Journal | | |
| 1. HAYAT – Journal of Faculty of Nursing and Midwifery [The] | | |
| 1. Health Information Management | | |
| 1. Hepatitis Monthly | | |
| 1. IBJ – Iranian Biomedical Journal | | |
| 1. IJCN – Iranian Journal of Child Neurology | | |
| 1. IJEM – Iranian Journal of Endocrinology and Metabolism | | |
| 1. IJI – Iranian Journal of Immunology | | |
| 1. IJKD – Iranian Journal of Kidney Diseases | | |
| 1. IJME – Iranian Journal of Medical Education | | |
| 1. IJMS – Iranian Journal of Medical Sciences | | |
| 1. IJPR – Iranian Journal of Pharmaceutical Research | | |
| 1. International Journal of Endocrinology and Metabolism | | |
| 1. International Journal of Environmental Research | | |
| 1. International Journal of Environmental Science and Technology | | |
| 1. Iran Journal of Nursing | | |
| 1. Iranian Journal of Allergy, Asthma and Immunology | | |
| 1. Iranian Journal of Basic Medical Sciences | | |
| 1. Iranian Journal of Clinical Infectious Diseases | | |
| 1. Iranian Journal of Dermatology | | |
| 1. Iranian Journal of Diabetes and Lipid Disorders | | |
| 1. Iranian Journal of Environmental Health Science and Engineering | | |
| 1. Iranian Journal of Epidemiology | | |
| 1. Iranian Journal of Fertility and Sterility | | |
| 1. Iranian Journal of Medical Microbiology | | |
| 1. Iranian Journal of Nuclear Medicine | | |
| 1. Iranian Journal of Nursing and Midwifery Research [IJNMR] | | |
| 1. Iranian Journal of Nutrition Sciences and Food Technology | | |
| 1. Iranian Journal of Obstetrics, Gynecology and Infertility [The] | | |
| 1. Iranian Journal of Otorhinolaryngology | | |
| 1. Iranian Journal of Parasitology | | |
| 1. Iranian Journal of Pediatrics | | |
| 1. Iranian Journal of Psychiatry and Clinical Psychology | | |
| 1. Iranian Journal of Public Health | | |
| 1. Iranian Journal of Radiation Research | | |
| 1. Iranian Journal of Radiology | | |
| 1. Iranian Journal of Reproductive Medicine | | |
| 1. IRCMJ – Iranian Red Crescent Medical Journal | | |
| 1. JAMC – Journal of Ayub Medical College – Abbotabad – Pakistan | | |
| 1. JBMS – Journal of the Bahrain Medical Society | | |
| 1. JDT – Journal of Dentistry Tehran University of Medical Sciences | | |
| 1. JMS – Journal of Medical Sciences | | |
| 1. Journal of Arak University of Medical Sciences | | |
| 1. Journal of Babol University of Medical Sciences | | |
| 1. Journal of Clinical Laboratory [The] | | |
| 1. Journal of Dentistry – Tehran University of Medical Sciences | | |
| 1. Journal of Dentistry Shiraz University of Medical Sciences | | |
| 1. Journal of Gorgan University of Medical Sciences | | |
| 1. Journal of Hygiene and Health | | |
| 1. Journal of Isfahan Dental School | | |
| 1. Journal of Isfahan Medical School | | |
| 1. Journal of Islamic Dental Association of Iran [The] | | |
| 1. Journal of Kerman University of Medical Sciences | | |
| 1. Journal of Mashad Dental School | | |
| 1. Journal of Mazandaran University of Medical Sciences | | |
| 1. Journal of Medical Council of Islamic Republic of Iran | | |
| 1. Journal of Medical Education | | |
| 1. Journal of Medicinal Plants | | |
| 1. Journal of Qazvin University of Medical Sciences and Health Services [The] | | |
| 1. Journal of Shaheed Sadoughi University of Medical Sciences and Health Services | | |
| 1. Journal of Shahrekord University of Medical Sciences | | |
| 1. Journal of Tehran University Heart Center [The] | | |
| 1. Journal of the Arab Society for Medical Research | | |
| 1. Journal of the Egyptian National Cancer Institute | | |
| 1. Journal of the Egyptian Society of Obstetrics and Gynecology [The] | | |
| 1. Journal of the Egyptian Society of Parasitology | | |
| 1. Journal of Zanjan University of Medical Sciences and Health Services | | |
| 1. JPMI – Journal of Postgraduate Medical Institute | | |
| 1. JRMS – Journal of Research in Medical Sciences | | |
| 1. Kasr El Aini Journal of Surgery | | |
| 1. KOOMESH – Journal of the Samman University of Medical Sciences | | |
| 1. LJM – Libyan Journal of Medicine | | |
| 1. Mansoura Journal of Pharmaceutical Sciences | | |
| 1. Mansoura Medical Journal | | |
| 1. Medical Journal of Ahmed Maher Teaching Hospital [The] | | |
| 1. Medical Journal of Cairo University [The] | | |
| 1. Medical Journal of Reproduction & Infertility | | |
| 1. Medical Journal of Tabriz University of Medical Sciences and Health Services | | |
| 1. Medical Journal of Teaching Hospitals and Institutes [The] | | |
| 1. Medical Journal of the Islamic Republic of Iran | | |
| 1. Medical Sciences Journal of Islamic Azad University | | |
| 1. Middle East Journal of Anesthesiology | | |
| 1. New Egyptian Journal of Medicine [The] | | |
| 1. New Iraqi Journal of Medicine [The] | | |
| 1. Pakistan Journal of Medical Sciences | | |
| 1. Pakistan Journal of Pharmaceutical Sciences | | |
| 1. Pakistan Journal of Physiology | | |
| 1. Pan Arab Journal of Orthopaedic and Trauma [The] | | |
| 1. Pejouhandeh: Bimonthly Research Journal | | |
| 1. Quarterly Journal of Fundamentals of Mental Health [The] | | |
| 1. RMJ – Rawal Medical Journal | | |
| 1. Saudi Journal of Gastroenterology [The] | | |
| 1. Scientific Journal of Al–Azhar Medical Faculty [Girls] [The] | | |
| 1. Scientific Journal of Forensic Medicine | | |
| 1. Scientific Journal of Kurdistan University of Medical Sciences | | |
| 1. Scientific Medical Journal | | |
| 1. Shaheed Beheshti University Dental Journal | | |
| 1. Shaheed Beheshti University Journal of Faculty of Nursing & Midwifery | | |
| 1. Social Welfare Quarterly | | |
| 1. Strides in Development of Medical Education | | |
| 1. Suez Canal University Medical Journal | | |
| 1. Tanaffos | | |
| 1. Tanta Medical Sciences Journal | | |
| 1. Tehran University Medical Journal [TUMJ] | | |
| 1. Urology Journal | | |
| 1. Veterinary Medical Journal | | |
