# Supplementary material for: Bridging knowledge translation gap in health in developing countries: visibility, impact and publishing standards in journals from the Eastern Mediterranean
Source: BMC Med Res Methodol. 2012 May 11;12:66. doi: 10.1186/1471-2288-12-66 (PMC3430582; doi:10.1186/1471-2288-12-66)
Supplement: Additional file 3: Table S2 — Journals with more than 10 articles published in 2006 and cited in Web of Science (WoS) or SCOPUS during 2006-2009. [file 1471-2288-12-66-S3.doc]

**Table S2.** Journals with more than 10 articles published in 2006 and cited in Web of Science (WoS) or SCOPUS during 2006-2009*

| **Journal title** | **No. published articles†** | **No. articles (%) with citations** | **No. citations** | **No. self-citations (%)** |
| --- | --- | --- | --- | --- |
| **WoS:** | | | | |
| *Saudi Medical Journal* | 249 | 120 (48.2) | 297 | 90 (30.3) |
| *Eastern Mediterranean Health Journal* | 156 | 66 (42.3) | 126 | 17 (13.5) |
| *Journal of the College of Physicians and Surgeons Pakistan: JCPSP* | 139 | 58 (41.7) | 108 | 16 (14.8) |
| *Journal of the Pakistan Medical Association: JPMA* | 120 | 57 (47.5) | 131 | 13 (9.9) |
| *Medical Principles and Practice* | 87 | 56 (64.4) | 166 | 38 (22.9) |
| *Annals of Saudi Medicine* | 119 | 48 (40.3) | 108 | 24 (22.2) |
| *Pakistan Journal of Medical Sciences* | 119 | 39 (32.8) | 56 | 12 (21.4) |
| *International Journal of Environmental Science and Technology* | 68 | 37 (54.4) | 81 | 20 (24.7) |
| *Journal of the Egyptian Society of Parasitology* | 87 | 28 (32.2) | 39 | 0 (0.0) |
| *Pakistan Journal of Pharmaceutical Sciences* | 46 | 28 (60.9) | 55 | 18 (32.7) |
| *Journal of Ayub Medical College, Abbottabad: JAMC* | 68 | 25 (36.8) | 40 | 4 (10.0) |
| *Urology Journal* | 39 | 16 (41.0) | 30 | 4 (13.3) |
| *Tunisie Medicale, La* | 59 | 15 (25.4) | 17 | 0 (0.0) |
| *Middle East Journal of Anesthesiology* | 54 | 14 (25.9) | 18 | 0 (0.0 |
| *Iranian Biomedical Journal: IBJ* | 33 | 12 (36.4) | 22 | 13 (59.1) |
| *Archives of Iranian Medicine* | 22 | 11 (50.0) | 23 | 3 (13.0) |
| *DARU – Journal of Faculty of Pharmacy Tehran University of Medical Sciences* | 20 | 11 (55.0) | 16 | 10 (62.5) |
| *Neurosciences* | 76 | 11 (14.5) | 15 | 9 (60.0) |
| **SCOPUS:** | | | | |
| *Saudi Medical Journal* | 249 | 134 (53.8) | 370 | 95 (25.7) |
| *Eastern Mediterranean Health Journal* | 156 | 86 (55.1) | 169 | 33 (19.5) |
| *Journal of the College of Physicians and Surgeons Pakistan: JCPSP* | 139 | 72 (51.8) | 142 | 24 (16.9) |
| *Journal of the Pakistan Medical Association: JPMA* | 120 | 71 (59.2) | 179 | 23 (12.8) |
| *Medical Principles and Practice* | 87 | 57 (65.5) | 187 | 39 (20.9) |
| *Annals of Saudi Medicine* | 119 | 51 (42.9) | 131 | 29 (22.1) |
| *Pakistan Journal of Medical Sciences* | 119 | 46 (38.7) | 85 | 11 (12.9) |
| *International Journal of Environmental Science and Technology* | 68 | 39 (57.4) | 110 | 34 (30.9) |
| *Journal of the Egyptian Society of Parasitology* | 87 | 34 (39.1) | 56 | 0 (0.0) |
| *Journal of Ayub Medical College, Abbottabad: JAMC* | 68 | 31 (45.6) | 65 | 6 (9.2) |
| *Pakistan journal of pharmaceutical sciences* | 46 | 30 (65.2) | 70 | 21 (30.0) |
| *Tunisie Medicale, La* | 59 | 24 (40.7) | 26 | 1 (3.8) |
| *Urology Journal* | 39 | 21 (53.8) | 39 | 5 (12.8) |
| *Middle East Journal of Anesthesiology* | 54 | 16 (29.6) | 23 | 0 (0.0) |
| *Iranian Biomedical Journal: IBJ* | 33 | 15 (45.5) | 30 | 16 (53.3) |
| *DARU – Journal of Faculty of Pharmacy Tehran University of Medical Sciences* | 20 | 13 (65.0) | 20 | 10 (50.0) |
| *Journal of Medicinal Plants* | 44 | 12 (27.3) | 17 | 10 (58.8) |
| *Neurosciences* | 76 | 12 (15.8) | 15 | 9 (60.0) |
| *Journal of Research in Medical Sciences: JRMS* | 68 | 11 (16.2) | 16 | 6 (37.5) |
| *Saudi Pharmaceutical Journal: SPJ* | 30 | 11 (36.7) | 16 | 10 (62.5) |

*For WoS out of 85 journals and for SCOPUS out of 93 journals with articles published in 2006, which received citations during 2006-2009.

†No. of articles from the available tables of contents of the journals published in 2006.
