# Supplementary material for: Bridging knowledge translation gap in health in developing countries: visibility, impact and publishing standards in journals from the Eastern Mediterranean
Source: BMC Med Res Methodol. 2012 May 11;12:66. doi: 10.1186/1471-2288-12-66 (PMC3430582; doi:10.1186/1471-2288-12-66)
Supplement: Additional file 4: Table S3 — Editorial and publishing standards in Eastern Mediterranean journals (n=244). [file 1471-2288-12-66-S4.doc]

**Table S3.** Editorial and publishing standards in Eastern Mediterranean journals (n=244)

| **Evaluation item** | **No. of journals with missing item (%)** |
| --- | --- |
| **Bibliographical information on the cover page:** |  |
| Journal title | 0 (0.0) |
| ISSN | 65 (26.6) |
| Specification of the volume and issue number, and part number if appropriate | 3 (1.2) |
| Year of publication (plus the month of publication if the journal is a monthly, or the exact date of publication if the journal is a weekly) | 2 (0.8) |
| **Information about editorial structure:** |  |
| The name of the Editor-in-Chief, | 6 (2.5) |
| including affiliation where appropriate, and the town and country where he/she is currently located | 8 (3.3) |
| The names of the Editorial Board (or panel), etc. | 4 (1.6) |
| with countries where they are located | 7 (2.9) |
| The names of editors responsible for specific areas – e.g. Book Reviews Editor | 228 (93.4) |
| with the country where located | 228 (93.4) |
| The name of the editor or administrator responsible for handling receipt of submissions | 114 (46.7) |
| The correspondence address for the editorial office | 26 (10.7) |
| **Information about the publication within the journal:** |  |
| The name and address of the publisher | 119 (48.8) |
| The name of the organisation that sponsors or endorses the publication (if any ) | 56 (22.9) |
| If the journal is available online, this should be stated and the URL of the online version should be given | 42 (17.2) |
| The journal’s print-ISSN (and/or electronic-ISSN if online) | 197 (80.7) |
| The frequency of publication (monthly, bi-monthly, quarterly, etc.) | 98 (40.2) |
| The circulation (print/electronic) this information is rarely given in journals, but it is felt to be useful for authors, sponsors, advertisers, etc., so would prove to be a valuable addition to the journal information | 235 (96.3) |
| Information on subscription and single-issue prices, method of payment, etc. | 138 (56.6) |
| Copyright statement (indicating who owns the overall journal copyright) | 129 (52.9) |
| All bibliographical indexes and databases where the journal is listed | 136 (55.7) |
| **Information in the “Information for Authors” section:** |  |
| General information on the process of evaluating manuscripts, with a statement of the journal’s conformance with international editorial standards and a summary of the peer review process | 108 (44.3) |
| Guidance on how the journal manages conflicts of interest between referee and author, referee and research sponsor, author and research sponsor, etc. | 162 (66.4) |
| A clear statement of expectations regarding ethical conduct in clinical and animal research | 161 (66.0) |
| Requirements regarding observance of the patient’s privacy rights and confidentiality of medical information | 166 (68.0) |
| Instructions regarding assignment of copyright or license-to-publish | 110 (45.1) |
| The postal address, website and e-mail address for submission or articles (as appropriate), and contact details for any enquiries (telephone, Email, postal) | 48 (19.7) |
| Clear guidance about the preparation of references (this is one of the most difficult items for authors to prepare correctly, and can undermine the credibility of a good article) | 64 (26.2) |
| A list of the types of articles the journal seeks to publish | 66 (27.1) |
| A clear statement of the aims and scope (or remit) of the journal | 100 (40.7) |
| **Composition of the first page of published articles:** |  |
| The full title of the article. This should be no longer than is necessary to convey the gist of the article, while avoiding vagueness or incompleteness, or promising more than the article actually delivers. Journals which publish in local languages should also give the title in English. | 0 (0.0) |
| The names of all the authors. Whether full first names or simply initials are given depends on the taste and judgment of the editors, but whatever policy is adopted should be applied consistently wherever possible (note: some authors will wish to be listed by their initials only even if full first names are generally given; in the Anglo-Saxon tradition such wishes are always respected). | 2 (0.8) |
| The exact contribution of each co-author, preferably in the following categories: (1) study design: (2) data collection: (3) statistical analysis: (4) literature search: (5) funds collection. No-one should be listed as a co-author who has not made a significant contribution to the work. E.g. the practice of automatically including heads of departments where the research was done (when they were not directly involved in the research), is to be actively discouraged. | 232 (95.1) |
| The institutional affiliation of each author, if any. Authors without a formal affiliation (e.g. working exclusively in a private practice) should give their city of residence. Academic titles and positions, such as “Prof.” or “Department Head,” is to be discouraged. At a minimum, the town and country of each author should be given, as this provides valuable information about the source of the article. | 11 (4.5) |
| Sources of financial support. The name of the supporting institution and grant number should be given. One of the following three headings should be used: [I] “Supported in part by” + name of the supporting institution and grant number [ii] "Departmental sources” – for research supported solely by university/hospital/employment funds [iii] “Self financing” – for research financed privately by authors (It is not common for this to appear on the opening page, but it is felt to be a useful – and sometimes crucial – piece of information before users read the article.) | 226 (92.6) |
| Precise bibliographic reference of the article (the name of the journal, year and volume number, page numbers), so that all copies of the article will contain complete bibliographic information even when detached from the rest of the journal. | 40 (16.4) |
| The dates when the manuscript was received in the editorial office and when it was accepted for publication. This gives potential authors some idea of the lead time for publication in the journal. | 151 (61.9) |
| The URL address or DOI (Digital Object Identifier) to the online version [if available]. | 20 (8.2) |
| A correspondence address for one of the authors, preferably with an e-mail address. | 66 (27.0) |
| A summary of the article (abstract) of 200-250 words. The summary should summarise the main points/findings of the article – it should not be confused with an Introduction. Within medical journals the abstract should be structured, and follow the structure of the article with the exception of the Discussion (Background, Material and methods, Results, Conclusions). An English summary should always be provided for articles published in another language, since frequently only these are indexed by international databases. If an English summary is given, a summary in the local language is optional. | 6 (2.5) |
| 3-6 key words, which should not be words that also occur in the title of the article. (For biomedical articles key words from the MeSH catalogue should be used). Key words should not be adjectives, and should not repeat the title of the article. English key words should always be provided for articles published in another language. | 19 (7.8) |
| **Structure of published original research articles on medical topics:** |  |
| Structured summary (200-250 words, as described above) | 1 (0.4) |
| Introduction (or Background). The purpose of the study should be given in the Introduction, not as a separate section. | 1 (0.4) |
| Materials and methods. The description should be sufficient to allow another researcher to duplicate the experiment. | 1 (0.4) |
| Results. Sufficient data should be given to allow an independent researcher to verify the results, including statistical analysis. All tables, graphs, photographs and figures should have legends in English (bi-lingual in journals published in other languages). | 1 (0.4) |
| Discussion. This should also include some remarks on the limitations of the study and suggestions for future research. | 1 (0.4) |
| Conclusions. Care should be taken not to present as “conclusions” statements that were not proven in the text. | 2 (0.8) |
| Acknowledgements (if appropriate). Acknowledgement should be regarded as a form of expressing the authors’ gratitude to those institutions or persons who enabled or facilitated the execution of the study, or otherwise made the study feasible, but did not make a personal contribution sufficient to justify co-authorship. | 1 (0.4) |
| Annex (if appropriate). The Annex may contain detailed descriptions of therapeutic and diagnostic techniques beyond the level of detail needed in the body of the article, samples of test forms and questionnaires used in the study, etc. | 0(0.0) |
| **References and notes:** |  |
| Notes should be kept to a minimum – and the content of the notes should not be extensive. Within a journal, use either footnotes or endnotes and retain a consistency of style. | 2 (0.8) |
| References should be grouped together at the end of the article (it is generally considered old-fashioned to put them in footnotes, and less useful to readers wishing to see the article sources). | 0 (0.0) |
| References should be presented either in Author/date sequence, or in consecutive order as they are cited in the text (Harvard or Vancouver styles respectively). Citations in the text should be either in the form of author/date or by Arabic numerals respectively. All authors should be listed in the reference, unless there are more than 6 authors, in which case the use of et al after the first 3 authors is acceptable. | 0 (0.0) |
| The references within a journal should all conform to the same style – particularly with regard to the order of the elements (title, volume, date etc.) and the style of the lettering (e.g. italic for journal tiles). | 0 (0.0) |
| **Abbreviations:** |  |
| Unless an abbreviation is extremely common, it should be described on the first mention within the text. Where abbreviations are used extensively a list of non-standard abbreviations used in the text should be provided either at the beginning of the article or at the end (before Acknowledgements and References). | 0 (0.0) |
| **Advertisements:** |  |
| Advertisements should be placed on editorial pages (at the beginning and/or at the end of the issue). The placement of advertisements within scientific content (i.e. directly before, inside, or directly after an article) implies a commercial bias in the article, which is to be avoided. | 0 (0.0) |
